# Supplementary material for: Quantum simulation of Hawking radiation and curved spacetime with a superconducting on-chip black hole
Source: Nat Commun. 2023 Jun 5;14:3263. doi: 10.1038/s41467-023-39064-6 (PMC10241825; doi:10.1038/s41467-023-39064-6)
Supplement: Supplementary file 1 — Supplementary Infomation [file 41467_2023_39064_MOESM1_ESM.pdf]

Supplementary Information for "Quantum simulation of Hawking radiation and curved spacetime with a superconducting on-chip black hole"

Yun-Hao Shi,[1,2,*] Run-Qiu Yang,[3,*] Zhongcheng Xiang,[1,*] Zi-Yong Ge,[4] Hao Li,[1,5] Yong-Yi Wang,[1,2] Kaixuan Huang,[6] Ye Tian,[1] Xiaohui Song,[1] Dongning Zheng,[1,2,7,†] Kai Xu,[1,2,6,7,8,‡] Rong-Gen Cai,[9,§] and Heng Fan[1,2,6,7,8,10,¶]

[1]Institute of Physics, Chinese Academy of Sciences, Beijing 100190, China
[2]School of Physical Sciences, University of Chinese Academy of Sciences, Beijing 100049, China
[3]Center for Joint Quantum Studies and Department of Physics, School of Science, Tianjin University, Tianjin 300350, China
[4]Theoretical Quantum Physics Laboratory, RIKEN Cluster for Pioneering Research, Wako-shi, Saitama 351-0198, Japan
[5]School of Physics, Northwest University, Xi'an 710127, China
[6]Beijing Academy of Quantum Information Sciences, Beijing 100193, China
[7]Songshan Lake Materials Laboratory, Dongguan, Guangdong 523808, China
[8]CAS Center for Excellence in Topological Quantum Computation, University of Chinese Academy of Sciences, Beijing 100049, China
[9]CAS Key Laboratory of Theoretical Physics, Institute of Theoretical Physics, Chinese Academy of Sciences, Beijing 100190, China
[10]Hefei National Laboratory, Hefei 230088, China

Supplementary Note 1. INTRODUCTION ON EDDINGTON-FINKELSTEIN COORDINATES

In this paper, the spacetime geometry is present by advanced Eddington-Finkelstein coordinates (AEFC) $\{t, x\}$. Though the coordinate "t" plays the role of "time" in this system, there are a few differences compared with the usual time coordinate. In this appendix, we will give a basic introduction.

One simple way to obtain an intuition of advanced Eddington-Finkelstein coordinates $\{t, x\}$ is to consider the wave propagating in flat spacetime. Let us consider a Minkowski spacetime. The usual Minkowski coordinates (MC) is $\{t_{\rm m}, x\}$, of which the metric reads

$$ds^2 = dt_{\rm m}^2 - dx^2 \tag{1}$$

The massless scalar field then will satisfy

$$\partial_{t_{\rm m}}^2 \phi - \partial_x^2 \phi = 0 \tag{2}$$

The solution of this equation, in general, is given by following "traveling wave solution",

$$\phi(t_{\rm m}, x) = \phi_1(t_{\rm m} + x) + \phi_2(t_{\rm m} - x)\,, \tag{3}$$

where $\phi_1(t_{\rm m} + x)$ stands for the advanced solution and $\phi_2(t_{\rm m} - x)$ stands for the outgoing solution.

To covert the advanced Eddington-Finkelstein coordinates $\{t, x\}$, we consider a coordinates transformation

$$t = t_{\rm m} + x\,, \tag{4}$$

and so the metric then reads

$$ds^2 = dt^2 - 2dtdx\,. \tag{5}$$

It needs to note that, though $dt_{\rm m} \neq dt$, we still have

$$\partial_{t_{\rm m}} h|_{\rm MC} = \partial_t h|_{\rm AEFC}\,, \tag{6}$$

for an arbitrary function h, i.e. the time derivatives are the same as that in the usual Minkowski coordinates and advanced Eddington-Finkelstein coordinates. Thus, the growth rate of a quantity in usual Minkowski coordinates can also be computed according to the time derivative of advanced Eddington-Finkelstein coordinates. On the contrary, if one wants to compute the spatial derivative, then the two coordinate systems will have different results

$$\partial_x h|_{\rm MC} \neq \partial_x h|_{\rm AEFC} \tag{7}$$

in general since the derivative in left-side fixes t_m but the derivative of right-side fixes $t = t_m + x$.

The propagators of wave are also very different in these two coordinate systems. From Eq. (3), one sees that the "traveling wave solution" in advanced Eddington-Finkelstein coordinates reads

$$\phi(t, x) = \phi_1(t) + \phi_2(t - 2x)\,, \tag{8}$$

This can also be obtained from the wave equation $2\partial_t \partial_x \phi + 2\partial_x^2 \phi = 0$. It is a little surprising that the advanced wave ϕ_1 has no propagator! In fact *the infalling mode now becomes a boundary condition rather than a propagator*. If we impose a boundary condition

$$\phi(t, \pm\infty) = 0\,, \tag{9}$$

then we have $\phi_1(t) = 0$ and there is only outgoing mode. Thus, the advanced Eddington-Finkelstein coordinates

* These authors contributed equally to this work.
† dzheng@iphy.ac.cn
‡ kaixu@iphy.ac.cn
§ cairg@itp.ac.cn
¶ hfan@iphy.ac.cn

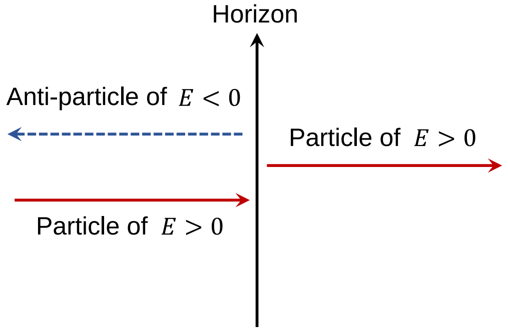

FIG. S1. The anti-particle flow of negative energy infalling toward the interior of the black hole can always be interpreted as a particle flow of positive energy outgoing from the interior.

with boundary condition (9) can only represent the propagator of outgoing modes. In other words, the advanced Eddington-Finkelstein coordinates play the role of a selector to choose only outgoing modes.

Though we assume that the spacetime is flat and the matter is a scalar field in the above discussion, the basic physical picture will still be true if we consider a curved 2-dimensional spacetime and Dirac field. In Fig. 2 of our main text, one can see that our model only simulates the outgoing modes, just as we expected in the above discussion. In general, the wave in gravitational fields contains both advanced modes and outgoing modes. The Hawking radiation is an energy flux towards infinity, i.e. carried by outgoing modes. This is one reason why this paper uses advanced Eddington-Finkelstein coordinates to study Hawking radiation.

Supplementary Note 2. MORE EXPLANATION ON THE TUNNELING PICTURE OF HAWKING RADIATION

This paper uses the picture of "quantum tunneling" to understand the Hawking radiation [1–3]. Though this is also a widespread picture to understand Hawking radiation in the community of black hole physics, it may not be familiar to other readers. Here we make a brief introduction to this picture.

At first glance, the tunneling picture is very different from the picture of "pair creation" outside the horizon. However, they are equivalent in physics. Based on the picture of "pair creation" in Hawking radiation, "particle-antiparticle pairs" can be created around the horizon. The antiparticle (negative energy) falls into the black hole and annihilates with positive energy particle inside the black hole, the particle outside the horizon is materialized and escapes into infinity. Note that the pair creation/annihilation is a virtual process, and the really materialized result is that the original particle inside the black hole disappears but an identical particle appears outside the horizon. The anti-particle of negative energy infalling the interior of the black hole can always be interpreted as a particle of positive energy outgoing from the interior, see schematic diagram Fig. S1. This leads to an equivalent picture to understand Hawking radiation via quantum tunneling: the particle inside the horizon escapes to the outside by quantum tunneling. Thus, the "tunneling picture" and "pair creation picture" are just two different pictures to understand the same physical phenomenon. Note that this "tunneling picture" does not violate causality since the spectrum is thermal and no information is carried.

Let us explain in detail how to use this tunneling picture to obtain the spectrum of radiation and corresponding temperature. For the spacetime with a black hole, the metric in the Schwarzschild coordinates $\{t_{\rm s}, x\}$ is given by $\mathrm{d}s^2 = f(x)\mathrm{d}t_{\rm s}^2 - f^{-1}(x)\mathrm{d}x^2$. We consider an outgoing mode with positive energy (corresponding to the observers of infinity) of a massless scalar or Dirac field, which can be written as ($\hbar = 1$)

$$\Phi(t_{\rm s}, x) \propto \exp\left[-i\omega\left(t_{\rm s} - \int \frac{\mathrm{d}x}{f(x)}\right)\right]. \tag{10}$$

By using the Eddington-Finkelstein coordinates we have

$$\Phi(t, x) \propto \exp\left[-i\omega\left(t - 2\int \frac{\mathrm{d}x}{f(x)}\right)\right]. \tag{11}$$

Since $f(x)$ has a root at $f(x_{\rm h})$, we separate integration into two parts,

$$\int \frac{\mathrm{d}x}{f(x)} = F(x) + \int \frac{\mathrm{d}x}{2g_{\rm h}(x-x_{\rm h})} = F(x) + \frac{1}{2g_{\rm h}}\ln|x-x_{\rm h}|, \tag{12}$$

where

$$F(x) = \int \left[\frac{1}{f(x)} - \frac{1}{2g_{\rm h}(x-x_{\rm h})}\right]\mathrm{d}x \tag{13}$$

is regular at $x = x_{\rm h}$. Here $g_{\rm h} = f'(x_{\rm h})/2$ is the surface gravity. Thus, the outgoing positive mode then reads

$$\Phi(t, x) \propto e^{-i\omega[t-2F(x)]}|x-x_{\rm h}|^{i\omega/g_{\rm h}}. \tag{14}$$

This outgoing mode has an infinite number of oscillations as $x \to x_{\rm h}$ and therefore cannot be straightforwardly extended to the inner region from the region outside the horizon. As argued in ref. [1], we can use analytic continuation to connect two branches in a complex plane: the wave function Φ describing a particle state (positive

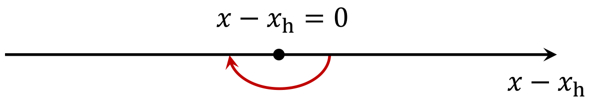

FIG. S2. The wavefunction are connected in the complex plane $\ln|x_{\rm h} - x| \to \ln|x_{\rm h} - x| - i\pi$ when x runs from outside horizon into inside horizon, which yields $|x_{\rm h} - x|^{i\omega/g_{\rm h}} \to |x_{\rm h} - x|^{i\omega/g_{\rm h}} e^{\pi\omega/g_{\rm h}}$.

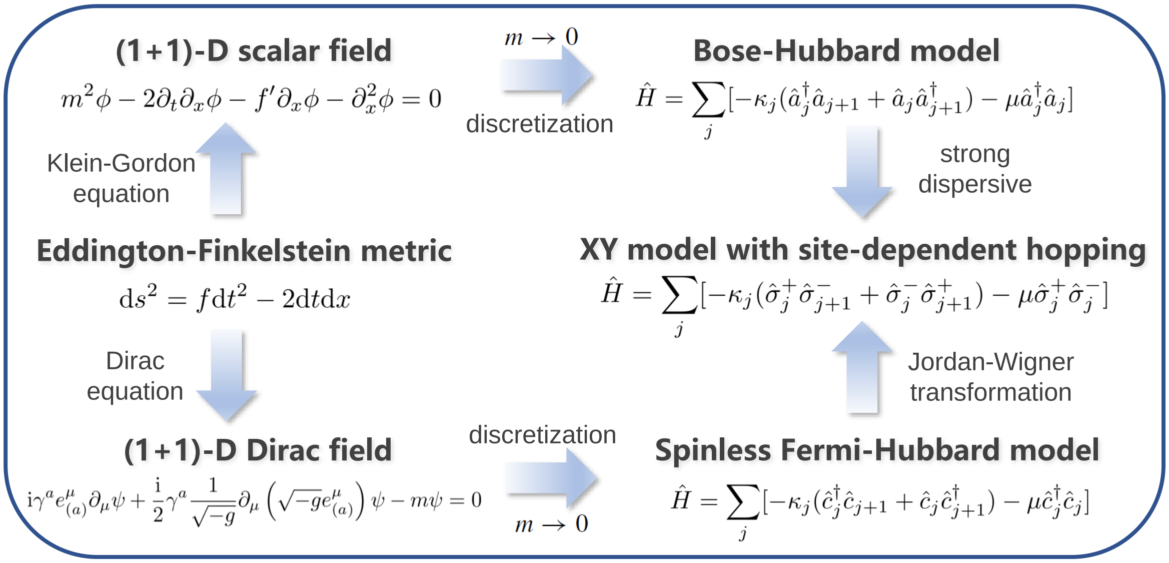

FIG. S3. The theoretical framework of simulating quantum field theory in (1+1)-D curved spacetime with quantum many-body systems. The "time" t in the infalling Eddington-Finkelstein metric is given by $t = t_\mathrm{s} + \int f^{-1}\mathrm{d}x$ where $\{t_\mathrm{s}, x\}$ are the Schwarzschild coordinates. In (1+1)-D configurations, the massless scalar field ϕ and the massless Dirac field ψ can be discretized into the Bose-Hubbard model and the spinless Fermi-Hubbard model respectively, where the static curved spacetime background is encoded into the site-dependent hopping coupling distribution κ_j satisfying Eq. (41). Here the on-site potential μ is an arbitrary constant. Note that both the Bose-Hubbard model and the spinless Fermi-Hubbard model can be transformed into the XY model, which implies their equivalence in (1+1)-D spacetime. This schematic diagram briefly summarizes the theory in ref. [4].

frequencies) can be analytically continued to a complex plane (see Fig. S2). Then we obtain

$$\Phi(t,x) = \begin{cases} e^{-i\omega[t-2F(x)]}|x-x_\mathrm{h}|^{i\omega/g_\mathrm{h}}, & x > x_\mathrm{h} \\ e^{-i\omega[t-2F(x)]}|x_\mathrm{h}-x|^{i\omega/g_\mathrm{h}}e^{\pi\omega/g_\mathrm{h}}, & x < x_\mathrm{h}\,. \end{cases} \tag{15}$$

This gives us the tunneling rate

$$P = e^{-2\pi\omega/g_\mathrm{h}}, \tag{16}$$

which is identical to the detailed balance relation for transition rates in a thermal environment [5]. Note that the tunneling rate (16) stands for the rate of a single particle. It is possible that the tunneling of multiple particles happens simultaneously. For bosons we have

$$\begin{array}{rccccc} \text{particle number:} & 0 & 1 & 2 & 3 & \cdots \\ \text{probability:} & 1 & P & P^2 & P^3 & \cdots \end{array} \tag{17}$$

Thus the occupation number of energy ω reads

$$n(\omega) = \frac{\sum_{k=0}^{\infty} kP^k}{\sum_{k=0}^{\infty} P^k} = \frac{1}{e^{2\pi\omega/g_\mathrm{h}} - 1}\,. \tag{18}$$

This gives us the expected distribution of bosons and the temperature reads $T = g_\mathrm{h}/(2\pi)$ as predicted by Hawking. For fermions, if there is no other internal degree of freedom, the Pauli exclusion principle implies that there is at most one particle of same energy. Thus, Eq. (17) is replaced by

$$\begin{array}{rcc} \text{particle number } n: & 0 & 1 \\ \text{probability:} & 1 & P \end{array} \tag{19}$$

Thus the occupation number of energy ω reads

$$n(\omega) = \frac{\sum_{k=0}^{1} kP^k}{\sum_{k=0}^{1} P^k} = \frac{1}{e^{2\pi\omega/g_\mathrm{h}} + 1}\,. \tag{20}$$

This gives us the expected distribution of fermions and the temperature still reads $T = g_\mathrm{h}/(2\pi)$.

In addition, the composite system of the interior of the black hole and the exterior is isolated. The interior and the exterior exchange energy and particles via the horizon. Hence, the occupations Eq. (18) and (20) can be viewed as the statistical averages of grand canonical distributions concerning bosons and fermions, respectively.

Supplementary Note 3. MODEL AND HAMILTONIAN

Our experiment is performed on a superconducting quantum processor which consists of 10 transmon qubits ($Q_1 \sim Q_{10}$) and 9 transmon-type couplers ($C_1 \sim C_9$). Each qubit and coupler are frequency-tunable, but only qubits have the XY control line and the readout resonator. The total Hamiltonian of this type of all-transmon system, with N qubits and $N-1$ couplers,

can be expressed as

$$\hat{H} = \hat{H}_Q + \hat{H}_C + \hat{H}_{Q-Q} + \hat{H}_{Q-C} + \hat{H}_{C-C}, \quad (21)$$

$$\hat{H}_Q/\hbar = \sum_{j=1}^{N} \omega_{q_j} \hat{b}^\dagger_{q_j} \hat{b}_{q_j} + \frac{\alpha_{q_j}}{2} \hat{b}^\dagger_{q_j} \hat{b}^\dagger_{q_j} \hat{b}_{q_j} \hat{b}_{q_j}, \quad (22)$$

$$\hat{H}_C/\hbar = \sum_{j=1}^{N-1} \omega_{c_j} \hat{b}^\dagger_{c_j} \hat{b}_{c_j} + \frac{\alpha_{c_j}}{2} \hat{b}^\dagger_{c_j} \hat{b}^\dagger_{c_j} \hat{b}_{c_j} \hat{b}_{c_j}, \quad (23)$$

$$\hat{H}_{Q-Q}/\hbar = \sum_{j=1}^{N-1} g_{q_j,q_{j+1}} (\hat{b}^\dagger_{q_j} \hat{b}_{q_{j+1}} + \hat{b}_{q_j} \hat{b}^\dagger_{q_{j+1}}), \quad (24)$$

$$\hat{H}_{C-C}/\hbar = \sum_{j=1}^{N-1} g_{c_j,c_{j+1}} (\hat{b}^\dagger_{c_j} \hat{b}_{c_{j+1}} + \hat{b}_{c_j} \hat{b}^\dagger_{c_{j+1}}), \quad (25)$$

$$\hat{H}_{Q-C}/\hbar = \sum_{j=1}^{N-1} g_{q_j,c_j} (\hat{b}^\dagger_{q_j} \hat{b}_{c_j} + \hat{b}_{q_j} \hat{b}^\dagger_{c_j}) + g_{q_{j+1},c_j} (\hat{b}^\dagger_{q_{j+1}} \hat{b}_{c_j} + \hat{b}_{q_{j+1}} \hat{b}^\dagger_{c_j}), \quad (26)$$

where $\hbar$ is the reduced Planck constant (for convenience $\hbar$ will be assumed to be 1 in the following), $\hat{b}_{q_j} (\hat{b}_{c_j})$ and $\hat{b}^\dagger_{q_j} (\hat{b}^\dagger_{c_j})$ denote the annihilation and creation operators of the j-th qubit (coupler), respectively. The corresponding frequencies and anharmonicities are $\omega_{q_j} (\omega_{c_j})$ and $\alpha_{q_j} (\alpha_{c_j})$. Every pair of two neighbouring qubits and their middle coupler are coupled through exchange-type interactions with coupling strengths g_{q_j,c_j}, g_{q_{j+1},c_j} and $g_{q_j,q_{j+1}}$. Here, the total Hamiltonian has three parts, including qubit-qubit interaction $\hat{H}_{Q-Q}$, coupler-coupler interaction $\hat{H}_{C-C}$ and qubit-coupler interaction $\hat{H}_{Q-C}$. The total system is equivalent to a 19-qubit Bose-Hubbard model.

In our experiment, the strong dispersive condition $g_{q_j,c_j} \ll |\Delta_{q_j,c_j}|$ is satisfied, where $\Delta_{q_j,c_j} = \omega_{q_j} - \omega_{c_j}$ is the frequency detuning. By virtue of the so-called Schrieffer-Wolff transformation

$$\hat{U} = \exp\Big[\sum_{j=1}^{N-1} \frac{g_{q_j,c_j}}{\Delta_{q_j,c_j}} (\hat{b}^\dagger_{q_j} \hat{b}_{c_j} + \hat{b}_{q_j} \hat{b}^\dagger_{c_j}) + \frac{g_{q_{j+1},c_j}}{\Delta_{q_{j+1},c_j}} (\hat{b}^\dagger_{q_{j+1}} \hat{b}_{c_j} + \hat{b}_{q_{j+1}} \hat{b}^\dagger_{c_j}) \Big], \quad (27)$$

one can obtain the effective qubits Hamiltonian

$$\begin{aligned} \hat{\tilde{H}} &= \hat{U} \hat{H} \hat{U}^\dagger = \hat{\tilde{H}}_Q + \hat{\tilde{H}}_{Q-Q} \\ &= \sum_{j=1}^{N} \widetilde{\omega}_{q_j} \hat{b}^\dagger_{q_j} \hat{b}_{q_j} + \frac{\alpha_{q_j}}{2} \hat{b}^\dagger_{q_j} \hat{b}^\dagger_{q_j} \hat{b}_{q_j} \hat{b}_{q_j} \\ &+ \sum_{j=1}^{N-1} \widetilde{g}_{q_j,q_{j+1}} (\hat{b}^\dagger_{q_j} \hat{b}_{q_{j+1}} + \hat{b}_{q_j} \hat{b}^\dagger_{q_{j+1}}). \end{aligned} \quad (28)$$

with the corresponding dressed frequency

$$\widetilde{\omega}_{q_j} = \begin{cases} \omega_{q_j} + \frac{g^2_{q_j,c_j}}{\Delta_{q_j,c_j}}, & j = 1 \\ \omega_{q_j} + \frac{g^2_{q_j,c_j}}{\Delta_{q_j,c_j}} + \frac{g^2_{q_j,c_{j+1}}}{\Delta_{q_j,c_{j+1}}}, & 1 < j < N \\ \omega_{q_j} + \frac{g^2_{q_j,c_{j-1}}}{\Delta_{q_j,c_{j-1}}}, & j = N \end{cases} \quad (29)$$

and effective coupling strength

$$\widetilde{g}_{q_j,q_{j+1}} = g_{q_j,q_{j+1}} + \frac{g_{q_j,c_j} g_{q_{j+1},c_j}}{\Lambda^{c_j}_{q_j,q_{j+1}}}, \quad (30)$$

where $\Lambda^{c_j}_{q_j,q_{j+1}} = 2/\big(1/\Delta_{q_j,c_j} + 1/\Delta_{q_{j+1},c_j}\big)$ is the harmonic mean of the frequencies detuning between the j-th coupler and its nearest neighbor qubits. Eq. (30) implies that the effective qubit-qubit coupling is derived from their direct capacitive coupling and the indirect virtual exchange coupling via the coupler in between. If the frequency of coupler is above the frequencies of qubits, $\Lambda^{c_j}_{q_j,q_{j+1}} < 0$ holds so that the effective coupling $\widetilde{g}_{q_j,q_{j+1}}$ can be tuned from positive to negative monotonically with gradually decreasing the frequency of coupler. Experimentally, we use the arbitrary waveform generator (AWG) to generate various fast-bias voltages applied to the corresponding couplers. These pulses on the Z control lines change the frequencies of couplers and then make it possible for the superconducting circuit with tunable couplers to engineer an arbitrary coupling distribution.

With $\widetilde{g}_{q_j,q_{j+1}} \ll \alpha_{q_j}$, the effective Hamiltonian Eq. (28) can be rewritten as a site-dependent XY model:

$$\hat{\tilde{H}} = -\sum_{j=1}^{N-1} \kappa_j (\hat{\sigma}^+_j \hat{\sigma}^-_{j+1} + \hat{\sigma}^-_j \hat{\sigma}^+_{j+1}) - \sum_{j=1}^{N} \mu_j \hat{\sigma}^+_j \hat{\sigma}^-_j, \quad (31)$$

where $\hat{\sigma}^+_j$ $(\hat{\sigma}^-_j)$ is the raising (lowering) operator of the j-th qubit. Here we choose

$$\kappa_j = -\widetilde{g}_{q_j,q_{j+1}}, \quad \mu_j = -\widetilde{\omega}_{q_j}. \quad (32)$$

For the Hamiltonian Eq. (31), one can map the spin variables to spinless fermion operators by introducing the Jordan-Wigner transformation [6]: $\hat{\sigma}^+_j = \hat{c}^\dagger_j \exp\Big\{ \mathrm{i}\pi \sum_{l=1}^{j-1} \hat{c}^\dagger_j \hat{c}_j \Big\}$ and $\hat{\sigma}^-_j = \exp\Big\{ -\mathrm{i}\pi \sum_{l=1}^{j-1} \hat{c}^\dagger_j \hat{c}_j \Big\} \hat{c}_j$, where the operators $\hat{c}^\dagger$ and $\hat{c}_j$ satisfy the commutation relations of fermions, i.e., $\{\hat{c}_j, \hat{c}_k\} = \{\hat{c}^\dagger_j, \hat{c}^\dagger_k\} = 0$ and $\{\hat{c}_j, \hat{c}^\dagger_k\} = \delta_{jk}$. Hence, the effective Hamiltonian is mapped into a spinless fermion lattice model as

$$\hat{\tilde{H}} = -\sum_{j=1}^{N-1} \kappa_j (\hat{c}^\dagger_j \hat{c}_{j+1} + \hat{c}_j \hat{c}^\dagger_{j+1}) - \sum_{j=1}^{N} \mu_j \hat{c}^\dagger_j \hat{c}_j. \quad (33)$$

Supplementary Note 4. CORRESPONDENCE WITH TWO-DIMENSIONAL CURVED SPACETIME

Considering the Heisenberg equation $\mathrm{i}\frac{\mathrm{d}}{\mathrm{d}t}\hat{c}_j = [\hat{c}_j, \hat{\tilde{H}}]$, the evolution equation for the operator $\hat{c}_j$ can be given

by

$$\mathrm{i}\frac{\mathrm{d}}{\mathrm{d}t}\hat{c}_j = -\kappa_j\hat{c}_{j+1} - \kappa_{j-1}\hat{c}_{j-1} - \mu\hat{c}_j. \quad (34)$$

By introducing a variable transformation $\hat{\tilde{c}}_j(t) = (-\mathrm{i})^j e^{-\mathrm{i}\mu t}\hat{c}_j$, we obtain

$$\frac{\mathrm{d}}{\mathrm{d}t}\hat{\tilde{c}}_j(t) = -\kappa_j\hat{\tilde{c}}_{j+1}(t) + \kappa_{j-1}\hat{\tilde{c}}_{j-1}(t). \quad (35)$$

Here $\hat{\tilde{c}}_j(t)$ can be viewed as a quantized operator of a discrete field $\varphi_j(t)$, and the spatial position can be discretized as $x = x_j = jd - x_\mathrm{h}$, where $x_\mathrm{h} = j_\mathrm{h}d$ ($j \in \mathbb{Z}^+$) and d denotes the lattice constant. Note the factor $(-i)^j$ is important to obtain the correct Heisenberg equation. The similar trick is widely used in artificial lattices to simulate quantum fields in flat or curved spacetimes [7–9].

Now let us recover the continuous field $\varphi(t,x)$. If we define a function f that is dependent of the spatial position x_j and substitute κ_j as,

$$\kappa_j = \frac{f(x_{j+1}) + f(x_j)}{8d}, \quad (36)$$

according to Eq. (35), $\varphi(t,x_j) \to \hat{\tilde{c}}_j(t)/\sqrt{d}$ will obey the following relation in the continuum limit,

$$\begin{aligned}\frac{\partial}{\partial t}\varphi(t,x) &= -\frac{f(x_{j+1}) + f(x_j)}{8d}\varphi(t,x_{j+1}) + \frac{f(x_j) + f(x_{j-1})}{8d}\varphi(t,x_{j-1}) \\ &= -\frac{f(x_j)}{4}\cdot\frac{\varphi(t,x_{j+1}) - \varphi(t,x_{j-1})}{2d} - \frac{1}{4}\cdot\frac{f(x_{j+1})\varphi(t,x_{j+1}) - f(x_{j-1})\varphi(t,x_{j-1})}{2d} \\ &= -\frac{f(x)}{4}\cdot\frac{\partial}{\partial x}\varphi(t,x) - \frac{1}{4}\cdot\frac{\partial}{\partial x}\left[f(x)\varphi(t,x)\right] \\ &= -\frac{f(x)}{2}\cdot\frac{\partial}{\partial x}\varphi(t,x) - \frac{f'(x)}{4}\varphi(t,x). \end{aligned} \quad (37)$$

In fact, Eq. (37) can be considered as a special case of Dirac equation in the massless limit $m \to 0$ if we decompose the Dirac field operator into $\psi = \frac{1}{\sqrt{2}}(\xi+\varphi, \xi-\varphi)^\mathrm{T}$. In the light of refs. [10, 11], the Dirac equation in (1+1)-dimensional curved spacetime with the metric $g_{\mu\nu}$ is written as

$$\mathrm{i}\gamma^a e^\mu_{(a)}\partial_\mu\psi + \frac{\mathrm{i}}{2}\gamma^a\frac{1}{\sqrt{-g}}\partial_\mu\left(\sqrt{-g}e^\mu_{(a)}\right)\psi - m\psi = 0, \quad (38)$$

where the γ-matrices in the two-dimensional case are $\gamma^0 = \sigma_z$ and $\gamma^1 = \mathrm{i}\sigma_y$, and the dyad is chosen as

$$e^\mu_{(a)} = \begin{pmatrix} -1 & 1 \\ \frac{1-f}{2} & \frac{1+f}{2} \end{pmatrix}, \quad (39)$$

which satisfies the orthonormal condition $e^{(a)}_\mu e^\nu_{(a)} = \delta^\nu_\mu$. Thus, Eq. (38) can be decomposed into two independent equations,

$$\partial_t\varphi = -\frac{f}{2}\partial_x\varphi - \frac{f'}{4}\varphi + \frac{\mathrm{i}}{2}m\xi,\ \partial_x\xi = -\mathrm{i}m\varphi. \quad (40)$$

In the massless limit $m \to 0$, one can find that Eq. (40) is in accord with Eq. (37). Hence, what the effective Hamiltonian Eq. (33) describes is equivalent to a two-dimensional static curved spacetime governed by the massless Dirac equation if we set κ_j as

$$\kappa_j = \frac{f(x_{j+1}) + f(x_j)}{8d} \approx \frac{f(x_j + d/2)}{4d}. \quad (41)$$

There is only one single nondegenerate horizon x_h so that $f(x_\mathrm{h}) = 0$ and $f(x) > 0$ when $x > x_\mathrm{h}$ and

$$g_\mathrm{h} = \frac{1}{2}f'(x_\mathrm{h}) > 0, \quad (42)$$

where g_h is the surface gravity of the horizon, which gives the Hawking temperature $T_\mathrm{H} = g_\mathrm{h}/(2\pi)$. In the main text, we set $f(x) = \beta\tanh\eta x/\eta$ with corresponding Hawking temperature $T_\mathrm{H} = \beta/(4\pi)$ and

$$\kappa_j = \frac{\beta\tanh((j - j_\mathrm{h} + 1/2)\eta d)}{4\eta d}, \quad (43)$$

where η controls the scale of variation of f over each lattice site, which has the dimension of $1/d$. Here, we fix $j_\mathrm{h} = 3$, $\beta/(2\pi) = 4.39$ MHz, and $\eta d = 0.35$ in the analogue curved spacetime experiments.

What we have shown above is the correspondence between XY model and the (1+1)-D Dirac field. The case of scalar field governed by Klein-Gordon equation is similar. For a complete presentation, one can refer to the earlier theoretical work [4]. Here we briefly summarize the theoretical framework, as shown in Fig. S3.

Supplementary Note 5. EXPERIMENTAL SETUP AND DEVICE PARAMETERS

Our superconducting quantum processor is placed in a BlueFors dilution refrigerator. The base temperature of the mixing chamber (MC) is about 9 mK. All qubits

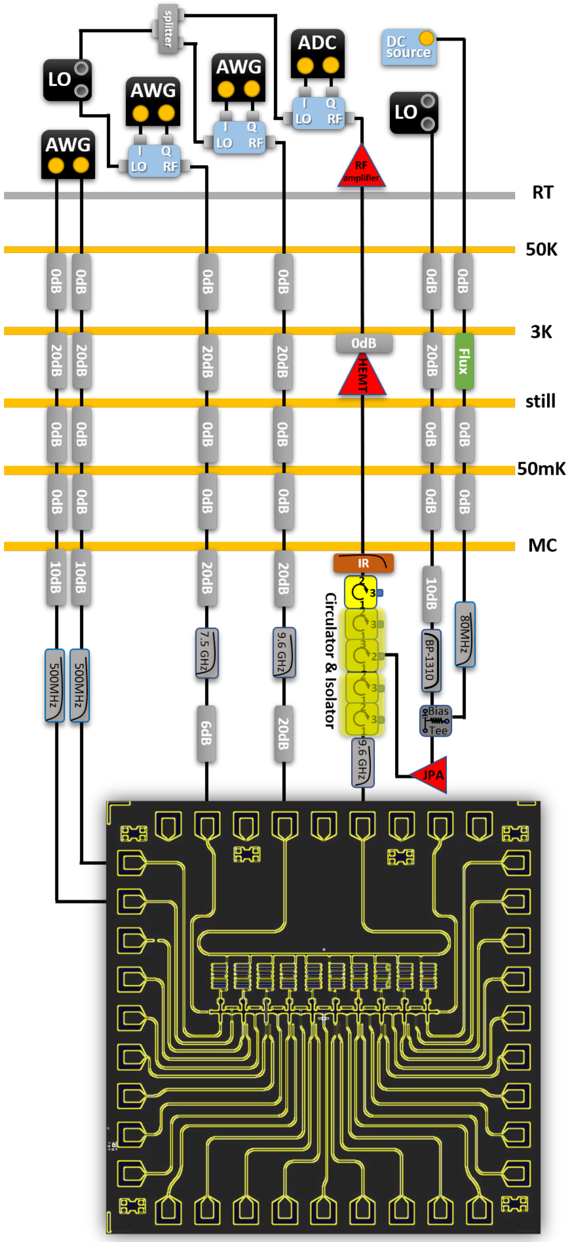

FIG. S4. A schematic diagram of the experimental system and partial wiring information.

share one readout line equipped with a Josephson parametric amplifier (JPA) and a high-electron-mobility transistor (HEMT). Pulse on the readout transmission line is first generated as a mixture of local oscillation (LO) and the envelopes from an arbitrary waveform generator (AWG) and then demodulated by an analog digital converter (ADC). In this experiment, we replace the DC bias with a long Z square pulse generated by AWGs. Both XY and Z control signals are programmed in advance before being uploaded to AWGs. A schematic diagram of experiment setup is given in Fig. S4.

The device parameters are briefly shown in Table S1.

All the parameters are characterized by various relatively efficient and automatic methods, especially the parameters concerning couplers. Details of those methods in our experiment will be introduced in the following.

Supplementary Note 6. EFFICIENT AND AUTOMATIC CALIBRATION FOR MULTI-QUBIT DEVICES WITH TUNABLE COUPLERS

Before carrying out our experiment for simulating an analogue black hole, we need to calibrate all 10 qubits and find the useful parameters of 9 couplers. This is far more difficult and time-consuming than calibrating a typical 10-qubit sample without tunable couplers. In order to measure and characterize device parameters more efficiently, we adopt an automatic calibration technology based on a combination of physical models and optimization methods.

A. Spectrum of qubit and frequency calibration

First and foremost, all the qubits are individually brought up through the standard single-qubit calibration (from identifying the readout resonator frequency to calibrating π pulse). If a qubit is brought up at a certain frequency, we need to perform a two-dimensional spectroscopy measurement to extract the mapping between Z-pulse amplitude (hereinafter referred as Zpa, each unit of 200 mV) of qubit bias and its frequency (such as Fig. S5) and this will contribute to automatically calibrate all the qubits together.

According to our all-transmon sample, each transmon consists of two parallel SIS-type Josephson junctions connected by a loop which is in series with a capacitor. The critical currents of two junctions are I_{c_1} and I_{c_2} and $E_{\rm C}$ denotes the charging energy of capacitor. By using the perturbation theory, the transition frequency can be approximately written as [12–14]

$$\omega(\Phi) \approx \sqrt{8E_{\rm JJ}E_{\rm C}\sqrt{\delta^2+\cos^2\left(\frac{\pi\Phi}{\Phi_0}\right)}}-E_{\rm C}, \qquad (44)$$

where $\Phi_0 = h/(2e)$ is the unit flux, $E_{\rm JJ} = \sqrt{I_{c_1}I_{c_2}}\Phi_0/\pi$ denotes the geometric mean of two junctions energy in the zero field and $\delta = |I_{c_1} - I_{c_2}|/(2\sqrt{I_{c_1}I_{c_2}})$ represents the junction asymmetry. Here, the total magnetic flux Φ is in direct proportion with the strength of the magnetic field threading the loop and this weak magnetic field induced by Z pulse is approximately proportional to Zpa ($\Phi \propto$ Zpa). Thus, the mapping between qubit Zpa and its frequency can be given by

$$\omega({\rm Zpa}) \approx \sqrt{8E_{\rm JJ}E_{\rm C}\sqrt{\delta^2+\cos^2\left(A\cdot{\rm Zpa}+\phi\right)}}-E_{\rm C} \qquad (45)$$

qubit	Q_1	Q_2	Q_3	Q_4	Q_5	Q_6	Q_7	Q_8	Q_9	Q_{10}
$\widetilde{\omega}_{q_j}^{01}/2\pi$ (GHz)	5.300	4.760	5.330	4.805	5.278	4.830	5.231	4.705	5.180	4.655
$\widetilde{\omega}_{q_j}^{r}/2\pi$ (GHz)	6.667	6.687	6.708	6.733	6.749	6.772	6.792	6.815	6.840	6.857
$E_{\rm C}/2\pi$ (MHz)	195.8	194.5	195.4	198.4	197.0	196.2	195.7	201.8	199.7	203.2
$E_{\rm JJ}/2\pi$ (GHz)	20.69	19.88	19.78	18.55	19.30	19.52	19.39	17.66	18.64	18.00
F_{0,q_j}	0.965	0.958	0.974	0.938	0.956	0.940	0.982	0.952	0.949	0.943
F_{1,q_j}	0.885	0.901	0.907	0.887	0.885	0.910	0.871	0.875	0.904	0.906
T_{1,q_j} (μs)	24.9	20.1	18.6	27.8	27.9	25.4	24.1	23.5	37.4	27.7
T_{2,q_j}^{*} (μs)	4.2	1.5	5.4	2.0	5.9	1.9	5.2	2.7	5.9	2.0
$T_{2,q_j}^{\rm Echo}$ (μs)	7.2	3.4	17.9	4.4	10.7	4.6	11.3	5.3	9.0	4.1

coupler	C_1	C_2	C_3	C_4	C_5	C_6	C_7	C_8	C_9
$g_{q_j,c_j}/2\pi$ (MHz)	98.07	84.13	98.88	85.96	96.46	87.50	96.00	85.63	96.02
$g_{q_{j+1},c_j}/2\pi$ (MHz)	85.72	96.68	88.64	97.46	85.36	96.78	85.89	97.40	83.36
$g_{q_j,q_{j+1}}/2\pi$ (MHz)	10.41	10.06	10.05	9.73	9.56	9.97	9.55	9.64	9.82

TABLE S1. List of device parameters. Here, $\widetilde{\omega}_{q_j(c_j)}^{01}$ is $|0\rangle\rightarrow|1\rangle$ transition frequency of the j-th qubit (coupler) with the corresponding readout frequency $\widetilde{\omega}_{q_j}^{r}$. $E_{\rm C}$ and $E_{\rm JJ}$ denote the charging energy and the Josephson energy. F_{0,q_j} and F_{1,q_j} are measure fidelities of $|0\rangle$ and $|1\rangle$, respectively. T_{1,q_j} represents the energy relaxation time of q_j at the idle point. The dephasing time T_{2,q_j}^{*} is characterized by the Ramsey fringe experiment, while $T_{2,q_j}^{\rm Echo}$ is measured by spin echo sequence with an inserted π pulse. The coupling strengths of exchange-type interactions between qubits and the corresponding coupler are g_{q_j,c_j} and g_{q_{j+1},c_j}, and the direct coupling of qubits is $g_{q_j,q_{j+1}}$.

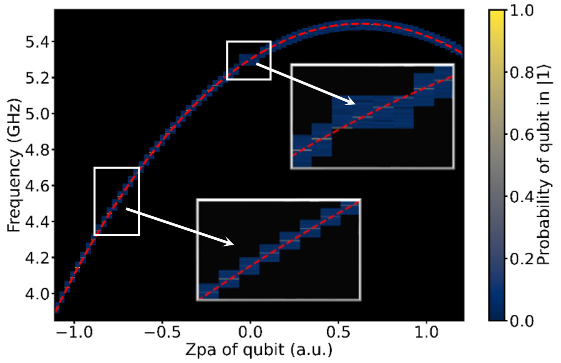

FIG. S5. Experimental data of qubit automatical spectroscopy measurement. Here we take the spectrum of Q_1 as an example. The black area is unscanned in order to save time. We first scan a small square area (about four columns data) around Zpa = 0 and then use polynomial curves to fit the peaks of these data. The corresponding polynomial fitting coefficients will help predict the next peak of the qubit spectrum. By constantly measuring, fitting and predicting, we obtain the experimental data of the qubit spectrum with a wide range. The mapping between qubit Zpa and its frequency can be obtained by fitting the experimental data based on Eq. (45).

and

$$\mathrm{Zpa}(\omega) \approx \frac{\arccos\left[\pm\sqrt{\frac{(\omega+E_{\rm C})^4}{(8E_{\rm JJ}E_{\rm C})^2}-\delta^2}\right]-\phi}{A}, \quad (46)$$

where $E_{\rm C}$ can be measured by the two-photon excitation experiment (double difference between two-photon excitation frequency and qubit frequency), the remaining parameters $E_{\rm JJ}$, δ, A and ϕ will be obtained by fitting the two-dimensional spectrum of qubits. Here parameter A describes the efficiency of qubit bias which depends on the attenuation on the Z control line, while ϕ is the initial flux shift. Even if the refrigerator temperature rises and cools again, only ϕ may have some displacement. As long as the circuit wiring does not change, parameter A will keep its value. Notice, however, that Eq. (45) and Eq. (46) need to be modified by the crosstalk of Z control lines if the multi-qubit case is involved.

When we design the multi-qubit levels, Eq. (46) will be beneficial to obtain the corresponding Zpa according to the target frequency. A more accurate frequency calibration can be implemented by the Ramsey fringe experiment.

B. Calibration of pulse distortion and Z crosstalk

Although the Z pulse generated by AWG is designed carefully, the shape of the pulse is distorted when it interacts with the qubit. To calibrate the distortion of step

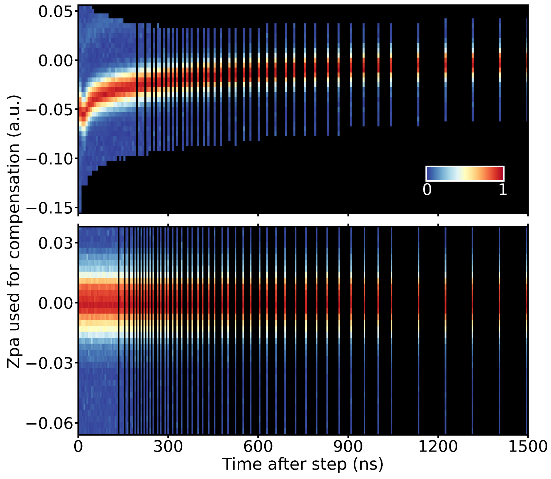

FIG. S6. Experimental data of pulse shape measurement. The black area is unscanned in order to save time. Here the heatmap denotes the probability of qubit in $|1\rangle$. The uncorrected pulse (up) is distorted, while the corrected result (down) shows a stationary step response.

response, we use several first-order infinite impulse response (IIR) filters and a finite impulse response (FIR) filter [15]. Here IIR filters are designed to be an integration of several exponential functions and the FIR filter is described by a polynomial with 20 parameters. The results of pre- and post-correction are shown in Fig. S6.

For the crosstalk of Z control lines between qubits and qubits or qubits and their non-nearest neighbor couplers, a routine Z crosstalk measurement with a small scanning range is adopted, which can be used to estimate the crosstalk coefficients by measuring the frequency response to the Z control lines. However, it may be better to extend to a wider range of scanning when it comes to the Z crosstalk of couplers to qubits. If the frequency of coupler approaches the frequency of qubit, the effect of anti-crossing will be amplified due to the strong coupling between the coupler and its nearest neighbor qubit, leading to a distinctly non-linear relationship between coupler Zpa and qubit Zpa (as shown in Fig. S7**b**). To correct the crosstalk from classical flux crosstalk of Z control lines that basically meet the linear relationship, we first select a range of data away from the resonance points to use linear fitting, and constantly fine-tune the corresponding crosstalk coefficient until a symmetrical anti-crossing pattern is obtained. For a more accurate Z crosstalk calibration, we still take advantage of Ramsey fringe experiment, but proximity to the resonance points should be avoided. Here we emphasize that in our procedure of calibration, Z crosstalk of couplers to qubits must be corrected in order to more accurately measure the spectrum of coupler and coupling strengths, as explained in the following.

C. Spectrum of coupler and anti-crossing of energy levels

As what mentioned above, it is difficult to directly excite and measure a coupler because it has no XY control line and readout resonator. Therefore, we make use of two qubits (Q_j and Q_{j+1}) that are adjacent to the coupler (C_j) to perform a coupler spectroscopy measurement (after the calibration of Z crosstalk). To be specific, we apply XY excitation pulse to one of the qubits (Q_j) and vary the Zpa of coupler. If the coupler is excited to $|1\rangle$ by the crosstalk from Q_j XY line, the frequency of another qubit (Q_{j+1}) will be changed due to the AC Stark effect between them. At the moment, a π pulse calibrated

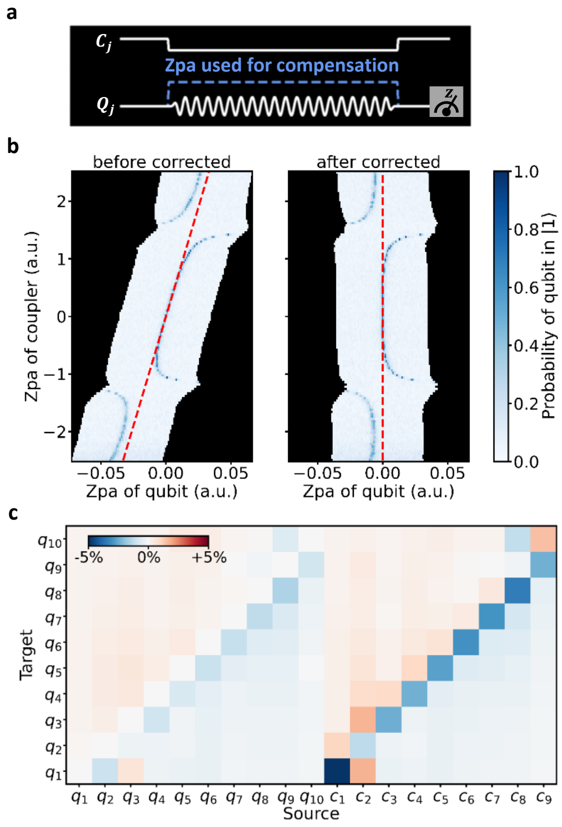

FIG. S7. Experimental data of automatical Z crosstalk calibration. **a**, Pulse sequence for measurement of Z crosstalk of coupler to qubit. **b**, Before Z crosstalk is corrected, one can observe a tilted anti-crossing pattern of qubit and its nearest neighbor coupler. By constantly fine-tuning the crosstalk coefficient and applying the Zpa to compensate for crosstalk from coupler Z line, a symmetrical anti-crossing pattern will be obtained after corrected. The black area is unscanned, while the red lines are the results of the linear fitting. Here we show the experimental data of Z crosstalk calibration **c**, All the coefficients of Z crosstalk. Compared with the high crosstalk from couplers Z line to qubits, the absolute coefficients of Z crosstalk between qubits are all at a low level ($< 2\%$).

before is unable to cause the perfect transition of Q_{j+1} due to its variation of frequency and its population of excited state will be decreased [16, 17] (see pulse sequence in the inset of Fig. S8**a**). In this way, one can obtain the spectrum of the local $Q_jC_jQ_{j+1}$ three-body system, which are actually the first three eigen-spectra (red lines in Fig. S8**a**) of the three-body Hamiltonian [16, 18]

$$\hat{H}_{Q_jC_jQ_{j+1}} = \sum_k \omega_k \hat{b}_k^\dagger \hat{b}_k + \frac{\alpha_k}{2}\hat{b}_k^\dagger \hat{b}_k^\dagger \hat{b}_k \hat{b}_k + \sum_{k\neq l} g_{k,l}(\hat{b}_k^\dagger \hat{b}_l + \hat{b}_k \hat{b}_l^\dagger) \tag{47}$$

with $k \in \{q_j, c_j, q_{j+1}\}$. In Eq. (47), the qubits frequencies ω_{q_j} and $\omega_{q_{j+1}}$ are fixed and all anharmonicities $\alpha = -E_C$ can be obtained by two-photon excitation measurement. Furthermore, the coupling between qubit and coupler is much stronger than $g_{q_j,q_{j+1}}$ in our device. Thus, there leaves 5 parameters to be determined in this three-body Hamiltonian, namely 2 coupling strengths (i.e., g_{q_j,c_j} and $g_{q_j,c_{j+1}}$) and 3 parameters of ω_{c_j} (i.e., E_{JJ}, A, ϕ in Eq. (45)).

Note that the smallest gaps of the two anti-crossing spectral lines represent twice the coupling strengths, respectively. However, it is inaccuracy to estimate the coupling strengths only by scanning the three-body spectrum due to the broadening of spectral lines and some impure peaks [19, 20]. For more accurate measurement, we scan two extra anti-crossing spectrums of two-body systems Q_jC_j and $Q_{j+1}C_j$, as shown in Fig. S8**b**. Truncated to two energy levels, the Hamiltonian of a qubit Q_j coupled to a coupler C_j can be expressed in the subspace basis $\{|10\rangle, |01\rangle\}$ as

$$\hat{H}_{Q_jC_j} = \begin{pmatrix} \omega_{q_j} & g_{q_j,c_j} \\ g_{q_j,c_j} & \omega_{c_j} \end{pmatrix}, \tag{48}$$

and its eigen-energy spectra are

$$\omega^{\pm}_{Q_jC_j} = \frac{\omega_{q_j} + \omega_{c_j}}{2} \pm \sqrt{g^2_{q_j,c_j} + \frac{(\omega_{q_j} - \omega_{c_j})^2}{4}}. \tag{49}$$

Similarly, the eigen-energy spectra of $Q_{j+1}C_j$ are

$$\omega^{\pm}_{Q_{j+1}C_j} = \frac{\omega_{q_{j+1}} + \omega_{c_j}}{2} \pm \sqrt{g^2_{q_{j+1},c_j} + \frac{(\omega_{q_{j+1}} - \omega_{c_j})^2}{4}}. \tag{50}$$

Combining the above two equations with the diagonalization result of Eq. (47), one can finally determine the coupling strengths between coupler and qubits (i.e., g_{q_j,c_j} and g_{q_{j+1},c_j}) and the mapping between coupler frequency ω_{c_j} and its Zpa. Actually, this is a multi-objective optimization problem of simultaneously fitting 3 spectroscopy results via 5 parameters. We utilize the optimization function `scipy.optimize.minimize` in the Python module SciPy to solve this problem.

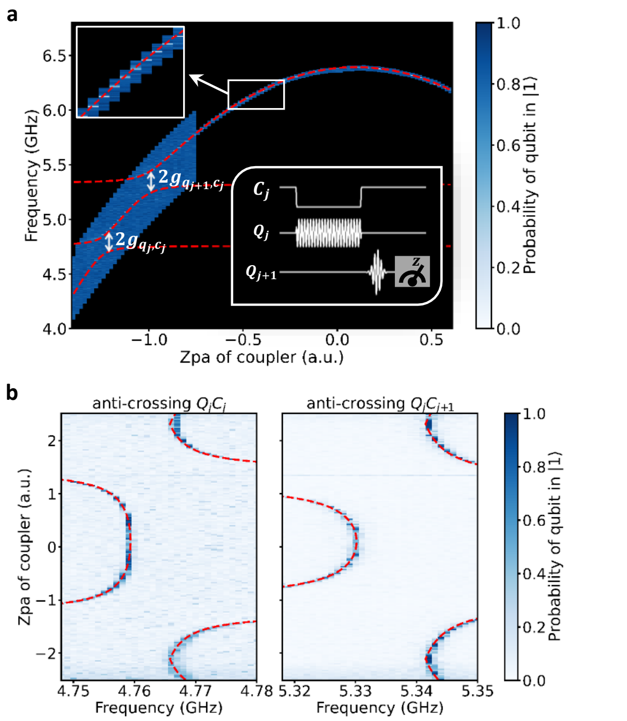

FIG. S8. Experimental data of coupler automatical spectroscopy measurement. The red curves are numerical simulation results for fitting the peaks of spectroscopy data, which is based on a multi-objective optimization. **a**, The spectrum of the local $Q_jC_jQ_{j+1}$ three-body system. The black area is unscanned, while the experimental data consists of the blue area. When the frequency of coupler is far from the qubits' frequency, we only need to scan a very narrow width like the single-qubit spectroscopy measurement. As it approaches the anti-crossing points, we increase the scan width to reduce the impact caused by predictive error, which ensures a clear three-body spectrum and saves time simultaneously. **b**, Experimental data of anti-crossing spectrums of Q_jC_j (left) and $Q_{j+1}C_j$ (right). Here the results of C_2 are taken as an example.

D. Measurement of the effective coupling

To measure the effective coupling strength $\widetilde{g}_{q_j,qj+1}$, we measure the joint probability as a function of qubit-qubit swapping time t and the Zpa of coupler [19, 20], as shown in Fig. S9**b**. Similar to Eq. (48), the swapping Hamiltonian of Q_jQ_{j+1} in the subspace effective basis $\{\,|\widetilde{10}\rangle, |\widetilde{01}\rangle\,\}$ is

$$\hat{H}_{Q_jQ_{j+1}} = \begin{pmatrix} \widetilde{\omega}_{q_j} & \widetilde{g}_{q_j,q_{j+1}} \\ \widetilde{g}_{q_j,q_{j+1}} & \widetilde{\omega}_{q_{j+1}} \end{pmatrix}. \tag{51}$$

If Q_jQ_{j+1} is initially in state $|\widetilde{01}\rangle$, the time-dependent joint probability $P_{01}(t) = \langle\widetilde{01}|\, e^{-i\hat{H}_{Q_jQ_{j+1}}t}\, |\widetilde{01}\rangle$ can be expressed as

$$P_{01}(t) = \frac{1}{2}\cos\left[\sqrt{4\widetilde{g}^2_{q_j,q_{j+1}} + (\widetilde{\omega}_{q_j} - \widetilde{\omega}_{q_{j+1}})^2}\, t\right] + \frac{1}{2}, \tag{52}$$

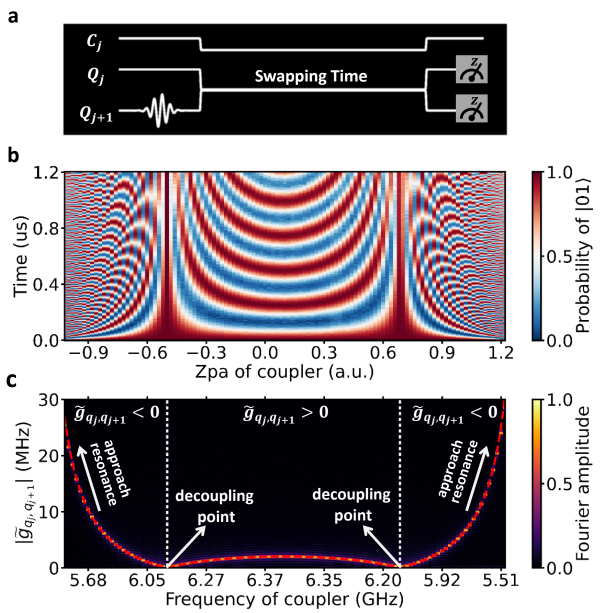

FIG. S9. Experimental data of the effective coupling strength measurement. **a**, Pulse sequence for measurement of swapping between qubits while changing the Zpa of coupler. **b**, Measured joint probability P_{01} of qubits vs Zpa of coupler (or corresponding frequency) and the swapping time. **c**, The Fourier transform of **b**, where the heatmap represents the normalized Fourier amplitude. The relation between absolute effective coupling strength $|\widetilde{g}_{q_j,q_{j+1}}|$ and coupler Zpa (or corresponding frequency) is given by each peak of normalized Fourier amplitude. The red dash line is the fitting curve of $|\widetilde{g}_{q_j,q_{j+1}}|$ by using Eq. (54), while white dot lines denote two decoupling points ($\widetilde{g}_{q_j,q_{j+1}} = 0$). As coupler frequency decreases, $\widetilde{g}_{q_j,q_{j+1}}$ decreases from positive to zero. Once it passes the decoupling point, $\widetilde{g}_{q_j,q_{j+1}}$ becomes negative and its absolute value will increase rapidly, especially approaching the resonance point of qubits.

which is reduced to

$$P_{01}(t) = \frac{1}{2}\cos\left(2\widetilde{g}_{q_j,q_{j+1}}t\right) + \frac{1}{2} \tag{53}$$

when the two qubits are resonant, namely $\widetilde{\omega}_{q_j} = \widetilde{\omega}_{q_{j+1}}$. Thus, the effective coupling strength can be calculated as half the Fourier frequency of probability $P_{01}(t)$. It needs to be emphasized that decoherence may cause the damping amplitude of swapping probability but does not affect the Fourier frequency.

For each Zpa of coupler (related to its frequency), we calculate $\widetilde{g}_{q_j,q_{j+1}}$ via measuring $P_{01}(t)$ and performing Fourier transform (as shown in Fig. S9**c**). Subsequently, one can utilize Eq. (30) to draw the mapping between the effective coupling strength and coupler Zpa:

$$\widetilde{g}_{q_j,q_{j+1}}(\mathrm{Zpa}) = g_{q_j,q_{j+1}} + \frac{g_{q_j,c_j}g_{q_{j+1},c_j}}{\omega_{c_j}(\mathrm{Zpa}) - \omega}, \tag{54}$$

where $\omega = \omega_{q_j} = \omega_{q_{j+1}}$ is the resonant frequency of qubits, the direct coupling $g_{q_j,q_{j+1}}$ is the fitted value and the coupler frequency $\omega_{c_j}(\mathrm{Zpa})$ obeys Eq. (45). Hence, if the Zpa of coupler is given, the effective coupling strength can be computed via Eq. (54); or given a target coupling, one can estimate the Zpa of coupler by

$$\mathrm{Zpa}(\widetilde{g}_{q_j,q_{j+1}}) \approx \frac{1}{A}\arccos\left[\pm\frac{1}{8E_{\mathrm{JJ}}E_{\mathrm{C}}}\sqrt{\left(\omega + E_C + \frac{g_{q_j,c_j}g_{q_{j+1},c_j}}{\widetilde{g}_{q_j,q_{j+1}} - g_{q_j,q_{j+1}}}\right)^2 - \left(8E_{\mathrm{JJ}}E_{\mathrm{C}}\delta\right)^2}\right] - \frac{\phi}{A}, \tag{55}$$

where E_{JJ} and E_{C} are the Josephson energy and the charging energy of coupler, respectively. Eq. (55) is a crucial foundation for engineering arbitrary coupling distribution in a superconducting circuit with tunable couplers.

Supplementary Note 7. ADDITIONAL DISCUSSION

For further discussion, we perform additional numerical simulations to compare and supplement with our results in this paper. In the following, the effects of disorders, different coupling distribution, finite size, and continuum limit are investigated.

A. The effects of disorders

In reality, qubits are doomed to be disturbed by various disorders, leading to the nuance between experimental conditions and theoretical assumptions. For a 1D-array of qubits, one can consider two disorders about next-nearest-neighbor (NNN) coupling g_{NNN} and on-site potential μ with the corresponding disorder strengths $W_{g_{\mathrm{NNN}}}$ and W_μ. Specifically, the Hamiltonian of disor-

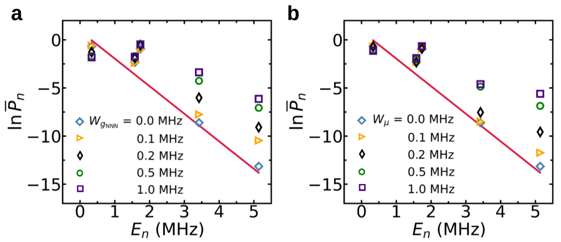

FIG. S10. The effects of two typical disorders on Hawking radiation. **a**, The logarithm of average radiation probability vs. the positive energy of particle with different disorder strengths of g_{NNN}. **b**, The logarithm of average radiation probability vs. the positive energy of particle with different disorder strengths of μ. Here, the red solid line represents the theoretical result.

ders is

$$\hat{H}_{\mathrm{dis}} = \sum_{j=1}^{8} g^{j}_{\mathrm{NNN}}(\hat{\sigma}^{+}_{j}\hat{\sigma}^{-}_{j+2} + \hat{\sigma}^{-}_{j}\hat{\sigma}^{+}_{j+2}) - \sum_{j=1}^{10}\mu_j\hat{\sigma}^{+}_{j}\hat{\sigma}^{-}_{j}, \quad (56)$$

where the site-dependent g^{j}_{NNN} and μ_j are in $[-W_{g_{\mathrm{NNN}}}, W_{g_{\mathrm{NNN}}}]$ and $[-W_{\mu}, W_{\mu}]$, and their distribution are assumed to be uniform. To probe the effects of disorders, we numerically model the dynamics of Hawking radiation by considering $\hat{H}_{\mathrm{dis}}$. In Fig. S10**a** and **b**, one can note that both disorders diverge the probability spectrum and theoretical results of Hawking radiation, especially

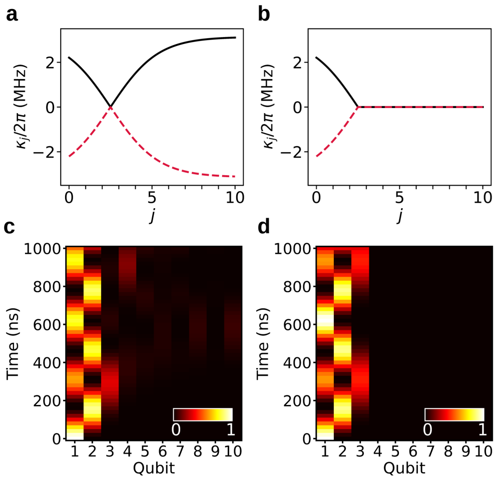

FIG. S11. Quantum walks of different coupling distribution. **a**, The couplings keep the same amplitude inside and outside of the black hole, where the black solid line represents all couplings keeping positive and the red dash line denotes all couplings remaining negative. These two cases are equivalent and the same results are shown in **c**. **b**, Non-zero coupling inside the black holes (black solid line and red dash line imply positive coupling and negative coupling respectively) and zero coupling between all sites outside the black hole, where the corresponding results of quantum walks are shown in **d**. Here we take the initial state $|1000000000\rangle$ as an example.

in the condition of strong disorder. However, we measure the NNN coupling of $g^{j}_{\mathrm{NNN}} \approx 0.1$ MHz and the frequencies difference of $|\mu_j - \omega_{\mathrm{ref}}| < 0.2$ MHz with reference frequency $\omega_{\mathrm{ref}}/(2\pi) \approx 5.1$ GHz. According to Fig. S10, such a small degree of disorders has little impact on the results of Hawking radiation in the experiment. In fact, we measure the initial density matrix of 7 qubits outside the horizon. The fidelity between the imperfect initial state in the experiment and the ideal initial state is 99.2% (see Fig. 3a in the main text), which may be caused by the XY crosstalk, thermal excitation, leakage, etc. We substitute such an experimental state for the ideal initial state in the numerical simulation of Hawking radiation, then the results of the numerical simulation agree with the experimental results better (see Fig. 3d in the main text).

B. Different coupling distribution

Admittedly, our model does not mandate flipping the sign of coupling κ_j near the horizon. In the main text, we request that the coupling goes monotonically from negative to positive (or vice versa) from the left of the horizon to its right side. This is based on the realistic consideration of the smoothness of $f(x)$. In fact, if instead of flipping the sign of couplings they were all kept positive (or negative) inside and outside of the black hole (Fig. S11**a**), all of the results would be similar, as shown in Fig. S11**c**. For the case with non-zero coupling inside the black hole but zero coupling between all sites outside the black hole (Fig. S11**b**), one can find the results inside the black hole are also similar to the results in case of flipping the sign of couplings, but it is quite different for the results outside the black hole. Due to the zero coupling between all sites outside, no particles can travel in the exterior (Fig. S11**d**) and thus no radiation can be detected by the observer outside.

C. The finite-size effects

Here we perform the numerical simulation of a 300-qubit chain to show the finite-size effects more clearly when we initialize the system by preparing a particle in the black hole. When the particle arrives at the horizon, it is going to be reflected back into the black hole in all probability but has a little chance to appear in the outside. The horizon is similar to a 'membrane' with certain transmittance, see Fig. S12. The probability of finding the particle outside P_{out} shows a general upward trend due to the Hawking radiation. However, the particle will be reflected when it arrives at the boundary (Q_1 or Q_{300}) due to the finite-size effects. When the particle reflected by the boundary of the black hole reaches the horizon again, it has a certain probability to escape into the outside and P_{out} thus increases again (see Fig. S12**a** and Fig. S12**b**). Conceivably, if there are no boundaries,

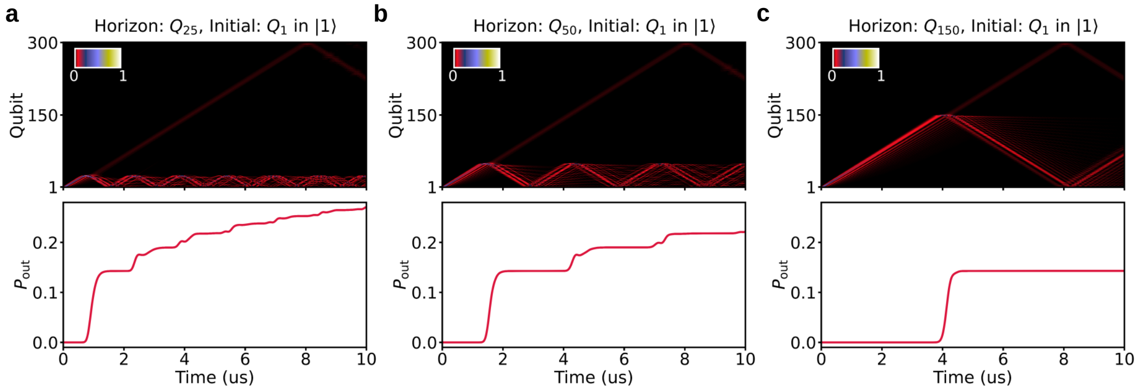

FIG. S12. Simulation of a 300-qubit chain with horizons at different locations. Here the coupling κ_j takes the form of Eq. (43), where $d = 0.35$ and $\beta/(2\pi) = 4.39$ MHz. From **a** to **c**, the corresponding horizons are located at Q_{25}, Q_{50} and Q_{150}, respectively. P_{out} is defined as the sum of probabilities of all the qubits outside the horizon.

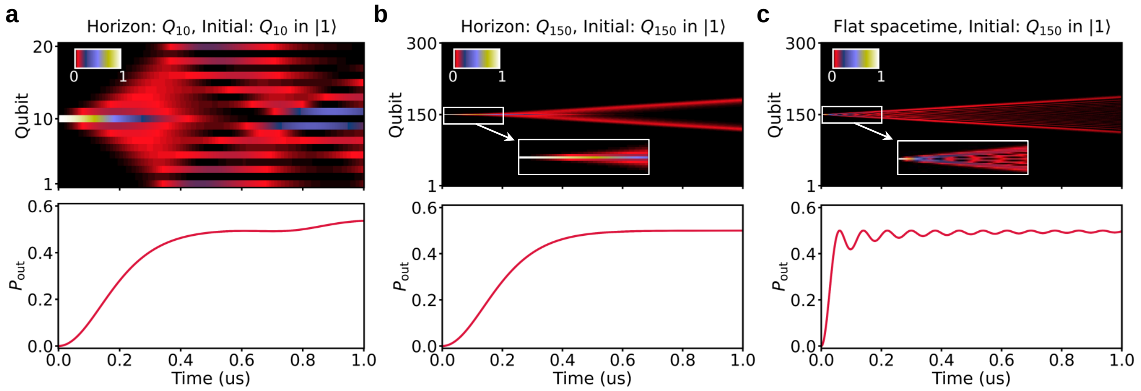

FIG. S13. The finite-size effects on the horizon. The particle is initialized at the horizon. **a**, Simulation of a 20-qubit chain with the horizon located at Q_{10}. **b**, Simulation of a 300-qubit chain with the horizon located at Q_{150}. Here the coupling κ_j takes the form of Eq. (43), where $d = 0.35$ and $\beta/(2\pi) = 4.39$ MHz. **c**, Simulation of a 300-qubit chain in flat spacetime with $\kappa_j = 3.14$ MHz. P_{out} is defined as the sum of probabilities of all the qubits outside the horizon.

P_{out} will increase to a certain value and eventually the particle reaches a steady state of radiation.

In addition, the finite size can also affect the horizon. In the continuous curved spacetime, the particle initialized at the horizon is bound to the horizon forever due to the zero couplings on both sides of the horizon. However, in the finite-size lattice, the coupling strengths on both sides of the horizon are not strictly zero even though they are very small. As shown in Fig. S13**a** and Fig. S13**b** (also Fig. 2**b** in the main text), although the particle seems to be localized at the horizon for a very short time, it is doomed to escape from the constraints. When the particle is far from the horizon, its behavior is similar to that in flat spacetime (see Fig. S13**b** and Fig. S13**c**).

D. Continuum limit

To confirm that our model realizes an analogy of black hole, we now consider the wave prorogating in classical and continuum limit. To simulate the wave prorogating in the classical limit, we consider a state $|\psi\rangle$ such that

$$\langle\psi|(\hat{c}_n - \psi_n)^\dagger(\hat{c}_n - \psi_n)|\psi\rangle \ll |\psi_n|^2 \tag{57}$$

where $\psi_n = \langle\psi|\hat{c}_n|\psi\rangle$. Then Eq. (34) becomes

$$-i\frac{\mathrm{d}}{\mathrm{d}t}\psi_n = \kappa_n\psi_{n-1} + \kappa_{n+1}\psi_{n+1} + \mu\psi_n \tag{58}$$

We choose the function $f(x)$ to be

$$f(x) = \tanh(\alpha x)\,.$$

with a constant α and set $\mu = 0$ for simplicity. This choice of metric describes an asymptotically flat spacetime since $f(x) \to 1$ as $x \to \pm\infty$.

In the asymptotically flat region $x \to \infty$, due to the translation symmetry, we can take a planar wave ansatz

$$\psi_{n,k} = c_0 \exp[-i(\omega_k t - 2x_n(k - \pi/4d)]\,, \tag{59}$$

Reference	Experimental system	Surface gravity $g_\mathrm{h}/(2\pi)$	Hawking temperature T_H	Ratio of mass M/M_s
[21]	Shallow water wave	$\sim 10^{-1}$ Hz	$\sim 10^{-12}$ K	$\sim 10^{4}$
[22–25]	Bose-Einstein condensates	$\sim 10^{1}$ Hz	$\sim 10^{-10}$ K	$\sim 10^{2}$
[26]	Bose-Einstein condensates	$\sim 10^{2}$ Hz	$\sim 10^{-9}$ K	$\sim 10^{1}$
This work	Superconducting qubits	$\sim 10^{5}$ Hz	$\sim 10^{-5}$ K	$\sim 10^{-3}$

TABLE S2. Comparison of analogue Hawking radiation with other experiments. Considering a Schwarzschild black hole in four-dimensional spacetime with the same Hawking temperature, the mass of analogue black hole can be calculated by $M/M_\mathrm{s} = 6.4\times 10^{-8}\mathrm{K}/T_\mathrm{H}$, where $M_\mathrm{s}\approx 2\times 10^{30}$ kg is the solar mass.

with $x_n = nd$. Note that a coefficient 2 of x_n appears in Eq. (59), which is different from the usual mode expansion $e^{-i(\omega_k t - xk+\varphi_0)}$. This is because here we use Eddington-Finkelstein coordinate and need Eq. (59) to match the equation (11). Eq. (59) leads to following dispersion relationship

$$\omega_k = \frac{1}{2d}\cos(2kd-\pi/2) = \frac{1}{2d}\sin 2kd\,. \qquad (60)$$

In the limit $d \ll 1$ but keep k finite, this recovers the dispersion relationship of massless particle and describes a massless particle travailing at the speed of light in the asymptotic flat regions. In the region of curved spacetime, we also require that the curvature radius of spacetime is much larger than the scale of wave packet so that the wave will propagate along trajectory of light. This requirement is easy found by using the Maxwell field in four-dimensional curved spacetime,

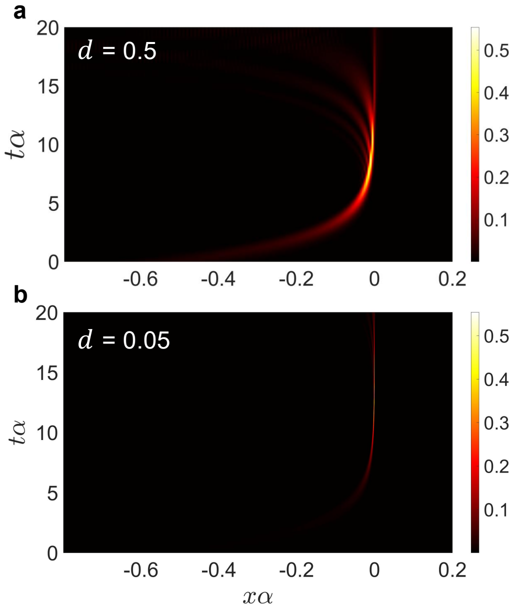

FIG. S14. Numerical results of outgoing modes with different widths of wave packets. Here we set $\Delta\alpha = 0.2$, $k = 0.01$, $\alpha = 0.01$, $d = 0.5$ (**a**) and $d = 0.05$ (**b**).

$$\Box A_\mu - R_{\mu\nu}A^\nu = 0\,. \qquad (61)$$

Here A_μ is the gauge field, $\Box$ is the d'Alembert operator in curved spacetime and $R_{\mu\nu}$ is the Ricci curvature tensor. In general, such an equation does not admit a plane wave solution due to the existence of the curvature term. In situations where the spacetime scale of variation of the electromagnetic field is much smaller than that of the curvature, the solutions of Maxwell's equations have a wave oscillating with nearly constant amplitude. In this case the *geometric optics approximation* can be used and eletromagnetic wave will travel along null geodesic approximately.

We can read the effective wavelength from Eq. (59) $\lambda = |2\pi/(2k-\pi/2d)| \approx d/4$. When the parameter α satisfies

$$\alpha\lambda \ll 1 \Leftrightarrow d\alpha \ll 1/4\,, \qquad (62)$$

The variation of spacetime caused by the curvature in curved region of spacetime will be much lower than the variation caused by phase factors of wave. Then the geometric optics approximation can be used. In this case, the wave will travel as light rays.

In numerical simulation, we take the initial wave packet to be Gaussian with the following form

$$\tilde{\psi}_n(0) = \frac{1}{Z}e^{-\frac{(x_n-\tilde{x}_0)^2}{\Delta^2}}\psi_{n,k}\,. \qquad (63)$$

Here Z is the normalized constant, Δ describes the width of wave packet, $\tilde{x}_0$ is the center of the initial wave packet and $\psi_{n,k}$ is defined by Eq. (59). Since the the wavepacket is modulated by Gaussian distribution, except Eq. (62) we also require $\Delta\alpha \ll 1$ so that geometric optical approximation is valid. On the other hand, to have a well-defined momentum, the width of wavepacket should much be larger than the wavelength, i.e. $\Delta \gg d$.

To summary, in order to check our model indeed forms an analogues of massless scalar particle in a black hole spacetime, we require the parameters to satisfy

$$kd \ll 1,\quad d\alpha \ll 1,\quad \Delta\alpha \ll 1,\quad \text{and } d/\Delta \ll 1 \qquad (64)$$

In the Fig. S14, we set parameters $\Delta\alpha = 0.2$, $k = 0.01$, $\alpha = 0.01$, $d = 0.5$ (**a**) and $d = 0.05$ (**b**) so that the geometric optical approximation is valid. From Fig. S14 we see that the outgoing mode inside the black hole will be increasingly closer to the horizon but not pass through

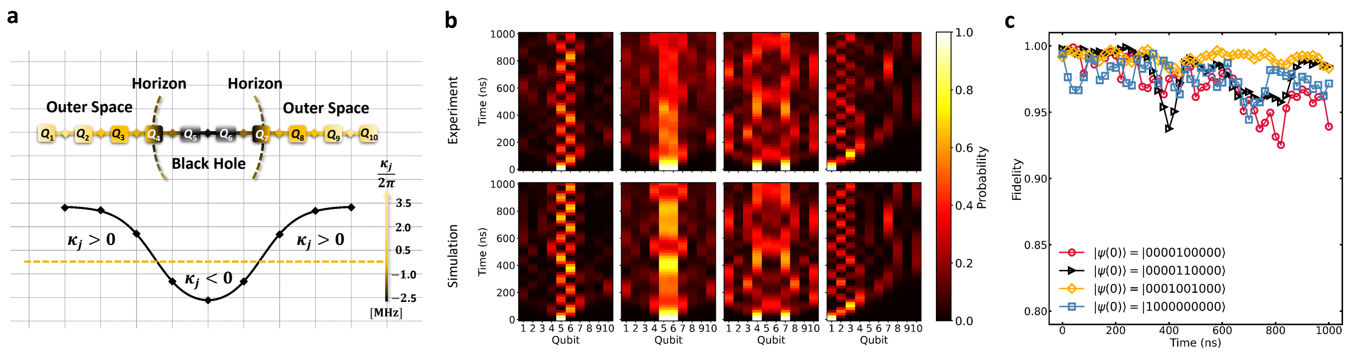

FIG. S15. Quantum walks in a 1D array of 10 superconducting qubits with black hole at the center. **a**, Schematic representation of the black hole at the center and the corresponding coupling distribution. **b**, Quantum walks in such a curved spacetime. The heatmap denotes the probabilities of excited-state for Q_i in time. The horizontal axis is indexed as qubit number i, the vertical axis is time. Here we show both the numerical simulation and experiment data. **c**, Fidelity of the experimental data compared to ideal numerical simulation of quantum walks.

the horizon. For finite d, the wave can only be "trapped" into the horizon for a finite time due to finite discretization. In the limit $d \to 0$, the "light" will be trapped into the horizon forever. This is just what we expect for the black hole horizon.

E. Comparison of measured Hawking temperature with other experiments

Here we compare the measured Hawking temperature with other previous experiments, as shown in Table S2. As we mentioned in the main text, the significant difference in magnitude of the results is attributed to the scales of the setup in different experimental systems. In our superconducting qubits system, we set a stronger surface gravity, contributing to a higher Hawking temperature.

Supplementary Note 8. EXTENDED DATA

In addition, we design an analogue black hole at the center of our 1D array superconducting qubits, see Fig. S15**a**. Here the coupling κ_j is designed as $\beta\left(\tanh((j-7/2)\eta d)-\tanh((j-13/2)\eta d)+1\right)/(4\eta d)$ with $\eta d = 1$ and $\beta/(2\pi) \approx 13.2$ MHz. The qubits Q_5 and Q_6 are in the interior of the black hole, Q_4 and Q_7 are at the horizon, and other qubits are in outer space. In Fig. S15**b**, we prepare four initial states to show quantum walks in such a curved spacetime, i.e., $|0000100000\rangle$, $|0000110000\rangle$, $|0001001000\rangle$ and $|1000000000\rangle$, respectively. The results of quantum walks of the particle at the center of the chain are similar to the results of the particle at the leftmost boundary in the main text. However, when the black hole is at the center, the finite size effect in the experimental 10-qubit 1D array becomes obvious, for which the tunneling out particle soon reaches the boundary and is reflected back.

[1] Thibaut Damour and Remo Ruffini, "Black-hole evaporation in the Klein-Sauter-Heisenberg-Euler formalism," Phys. Rev. D **14**, 332–334 (1976).

[2] Maulik K. Parikh and Frank Wilczek, "Hawking Radiation As Tunneling," Phys. Rev. Lett. **85**, 5042–5045 (2000).

[3] Michele Arzano, A. Joseph M Medved, and Elias C Vagenas, "Hawking radiation as tunneling through the quantum horizon," Journal of High Energy Physics **9**, 037–037 (2005).

[4] Run-Qiu Yang, Hui Liu, Shining Zhu, Le Luo, and Rong-Gen Cai, "Simulating quantum field theory in curved spacetime with quantum many-body systems," Phys. Rev. Research **2**, 023107 (2020).

[5] P. D. Nation, J. R. Johansson, M. P. Blencowe, and Franco Nori, "Colloquium: Stimulating uncertainty: Amplifying the quantum vacuum with superconducting circuits," Rev. Mod. Phys. **84**, 1–24 (2012).

[6] Elliott Lieb, Theodore Schultz, and Daniel Mattis, "Two soluble models of an antiferromagnetic chain," Annals of Physics **16**, 407–466 (1961).

[7] S. Longhi, "Klein tunneling in binary photonic superlattices," Physical Review B **81**, 075102 (2010).

[8] Felix Dreisow, Matthias Heinrich, Robert Keil, Andreas Tünnermann, Stefan Nolte, Stefano Longhi, and Alexander Szameit, "Classical simulation of relativistic zitter-

bewegung in photonic lattices," Phys. Rev. Lett. **105**, 143902 (2010).

[9] Christian Koke, Changsuk Noh, and Dimitris G. Angelakis, "Dirac equation in 2-dimensional curved spacetime, particle creation, and coupled waveguide arrays," Annals of Physics **374**, 162–178 (2016).

[10] J. S. Pedernales, M. Beau, S. M. Pittman, I. L. Egusquiza, L. Lamata, E. Solano, and A. del Campo, "Dirac Equation in $(1+1)$-Dimensional Curved Spacetime and the Multiphoton Quantum Rabi Model," Phys. Rev. Lett. **120**, 160403 (2018).

[11] R. B. Mann, S. M. Morsink, A. E. Sikkema, and T. G. Steele, "Semiclassical gravity in 1+1 dimensions," Phys. Rev. D **43**, 3948–3957 (1991).

[12] Jens Koch, Terri M. Yu, Jay Gambetta, A. A. Houck, D. I. Schuster, J. Majer, Alexandre Blais, M. H. Devoret, S. M. Girvin, and R. J. Schoelkopf, "Charge-insensitive qubit design derived from the Cooper pair box," Physical Review A **76**, 042319 (2007).

[13] P. Krantz, M. Kjaergaard, F. Yan, T. P. Orlando, S. Gustavsson, and W. D. Oliver, "A quantum engineer's guide to superconducting qubits," Applied Physics Reviews **6**, 021318 (2019).

[14] Sangil Kwon, Akiyoshi Tomonaga, Gopika Lakshmi Bhai, Simon J. Devitt, and Jaw-Shen Tsai, "Gate-based superconducting quantum computing," Journal of Applied Physics **129**, 041102 (2021).

[15] M. A. Rol, L. Ciorciaro, F. K. Malinowski, B. M. Tarasinski, R. E. Sagastizabal, C. C. Bultink, Y. Salathe, N. Haandbaek, J. Sedivy, and L. DiCarlo, "Time-domain characterization and correction of on-chip distortion of control pulses in a quantum processor," Applied Physics Letters **116**, 054001 (2020).

[16] Yuan Xu, Ji Chu, Jiahao Yuan, Jiawei Qiu, Yuxuan Zhou, Libo Zhang, Xinsheng Tan, Yang Yu, Song Liu, Jian Li, Fei Yan, and Dapeng Yu, "High-Fidelity, High-Scalability Two-Qubit Gate Scheme for Superconducting Qubits," Phys. Rev. Lett. **125**, 240503 (2020).

[17] Youngkyu Sung, Leon Ding, Jochen Braumüller, Antti Vepsäläinen, Bharath Kannan, Morten Kjaergaard, Ami Greene, Gabriel O. Samach, Chris McNally, David Kim, Alexander Melville, Bethany M. Niedzielski, Mollie E. Schwartz, Jonilyn L. Yoder, Terry P. Orlando, Simon Gustavsson, and William D. Oliver, "Realization of High-Fidelity CZ and ZZ-Free iSWAP Gates with a Tunable Coupler," Phys. Rev. X **11**, 021058 (2021).

[18] Fei Yan, Philip Krantz, Youngkyu Sung, Morten Kjaergaard, Daniel L. Campbell, Terry P. Orlando, Simon Gustavsson, and William D. Oliver, "Tunable Coupling Scheme for Implementing High-Fidelity Two-Qubit Gates," Phys. Rev. Applied **10**, 054062 (2018).

[19] X. Li, T. Cai, H. Yan, Z. Wang, X. Pan, Y. Ma, W. Cai, J. Han, Z. Hua, X. Han, Y. Wu, H. Zhang, H. Wang, Yipu Song, Luming Duan, and Luyan Sun, "Tunable Coupler for Realizing a Controlled-Phase Gate with Dynamically Decoupled Regime in a Superconducting Circuit," Physical Review Applied **14**, 024070 (2020).

[20] Huikai Xu, Weiyang Liu, Zhiyuan Li, Jiaxiu Han, Jingning Zhang, Kehuan Linghu, Yongchao Li, Mo Chen, Zhen Yang, Junhua Wang, Teng Ma, Guangming Xue, Yirong Jin, and Haifeng Yu, "Realization of adiabatic and diabatic CZ gates in superconducting qubits coupled with a tunable coupler," Chinese Physics B **30**, 044212 (2021).

[21] Silke Weinfurtner, Edmund W. Tedford, Matthew C. J. Penrice, William G. Unruh, and Gregory A. Lawrence, "Measurement of Stimulated Hawking Emission in an Analogue System," Phys. Rev. Lett. **106**, 021302 (2011).

[22] Oren Lahav, Amir Itah, Alex Blumkin, Carmit Gordon, Shahar Rinott, Alona Zayats, and Jeff Steinhauer, "Realization of a Sonic Black Hole Analog in a Bose-Einstein Condensate," Phys. Rev. Lett. **105**, 240401 (2010).

[23] M. Isoard and N. Pavloff, "Departing from Thermality of Analogue Hawking Radiation in a Bose-Einstein Condensate," Physical Review Letters **124**, 060401 (2020).

[24] Juan Ramón Muñoz de Nova, Katrine Golubkov, Victor I. Kolobov, and Jeff Steinhauer, "Observation of thermal Hawking radiation and its temperature in an analogue black hole," Nature **569**, 688–691 (2019).

[25] Victor I. Kolobov, Katrine Golubkov, Juan Ramón Muñoz de Nova, and Jeff Steinhauer, "Observation of stationary spontaneous Hawking radiation and the time evolution of an analogue black hole," Nature Physics **17**, 362–367 (2021).

[26] Jeff Steinhauer, "Observation of quantum Hawking radiation and its entanglement in an analogue black hole," Nature Physics **12**, 959–965 (2016).
